# Investigation of the Atypical Glass Transition and Recrystallization Behavior of Amorphous Prazosin Salts

**DOI:** 10.3390/pharmaceutics3030525

**Published:** 2011-08-25

**Authors:** Lokesh Kumar, Dharmesh Popat, Arvind K. Bansal

**Affiliations:** 1 Department of Pharmaceutics, National Institute of Pharmaceutical Education and Research (NIPER), S.A.S. Nagar, Mohali, Punjab-160 062, India; 2 Department of Pharmaceutical Technology (Formulations), National Institute of Pharmaceutical Education and Research (NIPER), S.A.S. Nagar, Mohali, Punjab-160 062, India

**Keywords:** prazosin, amorphous, glass transition, recrystallization

## Abstract

This manuscript studied the effect of counterion on the glass transition and recrystallization behavior of amorphous salts of prazosin. Three amorphous salts of prazosin, namely, prazosin hydrochloride, prazosin mesylate and prazosin tosylate were prepared by spray drying, and characterized by optical-polarized microscopy, differential scanning calorimetry and powder X-ray diffraction. Modulated differential scanning calorimetry was used to determine the glass transition and recrystallization temperature of amorphous salts. Glass transition of amorphous salts followed the order: prazosin mesylate > prazosin tosylate ∼ prazosin hydrochloride. Amorphous prazosin mesylate and prazosin tosylate showed glass transition, followed by recrystallization. In contrast, amorphous prazosin hydrochloride showed glass transition and recrystallization simultaneously. Density Functional Theory, however, suggested the expected order of glass transition as prazosin hydrochloride > prazosin mesylate > prazosin tosylate. The counterintuitive observation of amorphous prazosin hydrochloride having lower glass transition was explained in terms of its lower activation energy (206.1 kJ/mol) for molecular mobility at T_g_, compared to that for amorphous prazosin mesylate (448.5 kJ/mol) and prazosin tosylate (490.7 kJ/mol), and was further correlated to a difference in hydrogen bonding strength of the amorphous and the corresponding recrystallized salts. This study has implications in selection of an optimal amorphous salt form for pharmaceutical development.

## 1. Introduction

Modulation of solubility and/or dissolution rate remains a major challenge for successful development of many drug candidates. Various approaches are used to improve aqueous solubility, of which, salt formation and amorphous drug delivery are widely reported [1-3]. Recently, amorphous forms of drug salts have been evaluated to harness the advantages of both salt formation and amorphous drug delivery [4-7]. Amorphous salt forms increase the dissolution rate, via both a higher solubility of the ionized form relative to the unionized drug, as well as due to the higher apparent solubility of the amorphous form.

Properties of the amorphous salt forms, including the glass transition temperature (*T*_g_), are dictated by the type of counterion [4-7]. Higher *T*_g_ is generally a favorable property, since compounds with high *T*_g_ have a reduced ability to recrystallize at a given temperature, compared to the ones having lower *T*_g_. Tong and Zografi compared *T*_g_ of amorphous indomethacin free acid and its sodium salt, and found that amorphous indomethacin sodium salt had a higher *T*_g_ compared to amorphous indomethacin free acid. This was attributed to an increased electrostatic interaction of the counterion with the carboxylate group of indomethacin, thus leading to decreased molecular mobility and a consequent increase in *T*_g_ [6]. Tong *et al.* further evaluated the effect of counterion on the amorphous salts of indomethacin and observed that *T*_g_ of salts depend on the ionic density of counterion, which decreased in the order: Li^+^ > Na^+^ > K^+^ > Rb^+^ > Cs^+^. Smaller cation radius (and thus higher ionic density) of the counterion increased the electrostatic interaction between the drug and counterion. In contrast, larger cation radius decreased the ionic charge density, increased the molecular mobility and thus decreased the *T*_g_ of corresponding amorphous indomethacin salt [5].

Towler *et al.* reported *T*_g_ of amorphous salts of nicardipine and propranolol. Molecular descriptors including the p*K*_a_ and electrophilicity index of the counterion were observed to affect the value of *T*_g_. A direct correlation between the *T*_g_ and electrophilicity index was observed. Conversely, *T*_g_ decreased with a corresponding increase in p*K*_a_ of the counterion [7]. Guerrieri *et al.* similarly studied the effect of counterion on *T*_g_ of procaine salts. *T*_g_ of procaine salts was higher than the *T*_g_ of procaine free base. *T*_g_ varied considerably with the type of counterion, showing an increase with decrease in p*K*_a_ of the corresponding counterion [4].

**Figure 1.**
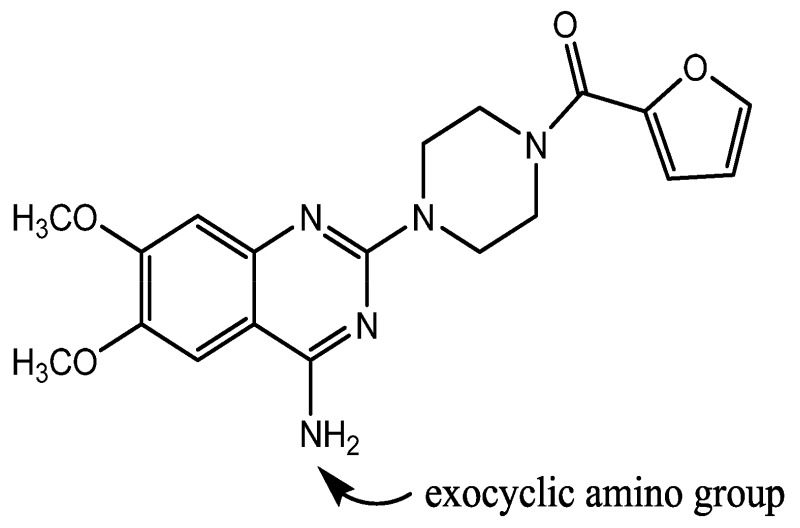
Structure of prazosin. Exocyclic amino group and N-1 quinazoline nitrogen are predominantly involved in intermolecular hydrogen bonding.

Effect of counterion on *T*_g_ of amorphous drug salts is evident from the aforementioned studies. However, no study has been reported on the effect of counterion on recrystallization behavior of amorphous salts. This manuscript aims to study the effect of counterion on glass transition and recrystallization behavior of amorphous salt forms, using prazosin as the model drug. Prazosin (Figure 1) is an α_1_ adrenergic blocker, used in the treatment of hypertension and benign prostatic hyperplasia [8,9]. Currently, anhydrous and polyhydrate form of hydrochloride salts are used commercially, and have a water content of less than 2% and 8–15% respectively [8]. Crystalline prazosin hydrochloride salt has a low aqueous solubility (1 mg/mL), contributing towards lower bioavailability (60%) [10]. Therefore, evaluation of amorphous salts of prazosin having higher solubility could help in selection of an amorphous salt for prazosin, having properties optimal for pharmaceutical development.

## 2. Experimental Section

### 2.1. Materials

Crystalline prazosin hydrochloride (PRB HCl) was purchased from Synthokem Laboratories, India and was used as supplied (chemical purity >99.9%). All other chemicals used were of analytical grade.

### 2.2. Methods

#### 2.2.1. Preparation of Crystalline Salts of Prazosin

Crystalline prazosin tosylate (PRB TSA) and prazosin mesylate (PRB MES) salts were prepared by reaction crystallization method. Briefly, prazosin free base (PRB) was first generated by dissolving PRB HCl in Ultrapure^®^ water, followed by alkalinization with 2.5 M sodium hydroxide, leading to precipitation of PRB. Obtained precipitate was collected by filtration, and characterized by techniques including DSC, TGA, ^1^H NMR and elemental analysis. PRB TSA and PRB MES salts were then prepared by dispersing PRB in acetonitrile:water (4:1; 20 mL), followed by addition of equimolar counterion dissolved in acetonitrile:water (4:1; 20 mL). In all cases, a white precipitate of the corresponding salt ensued immediately, which was filtered, dried and characterized by elemental analysis, ^1^H NMR, PXRD and DSC/TGA to confirm salt formation.

#### 2.2.2. Preparation of Amorphous Salts by Spray Drying

Prazosin salts degrade with melting, thus obviating use of melt quenching technique. Amorphous salts of prazosin were prepared by spray drying technique. Crystalline salts were dissolved in dimethyl formamide:water mixture (8:1), followed by spray drying using a laboratory spray dryer (Labultima, India). Parameters of spray drying were: atomization pressure: 0.7–0.9 kg/cm^2^, inlet temperature: 150–160 °C, outlet temperature: 65–70 °C, feed rate: 3 mL/min and aspirator volume: 100 mm/WC. Sample was thereafter stored over phosphorous pentoxide in vacuum desiccator.

#### 2.2.3. Optical and Polarized Light Microscopy

Particle characteristics were assessed by optical microscopy using Leica DMLP polarized light microscope (Leica Microsystems, Germany). Photomicrographs were obtained using RICOH XR-X3000D camera (RICOH, Japan). Photomicrographs were processed using Linksys^®^ 32 (version 1.8.9) software.

#### 2.2.4. Powder X-ray Diffractometry (PXRD)

PXRD patterns of samples were recorded at room temperature on Bruker's D8 advance diffractometer (Bruker, Germany) with CuKα radiation (1.54 Å), at 40 kV, 40 mA passing through nickel filter. Analysis was performed in continuous mode with a step size of 0.01° and step time of 1 second over an angular range of 3–40° 2*θ*. Diffractograms were analyzed with DIFFRAC^plus^ EVA (version 9.0) diffraction software.

#### 2.2.5. Modulated Differential Scanning Calorimetry (MDSC)

MDSC analysis was conducted using DSC, Model Q2000 (TA Instruments, USA). DSC cell was constantly purged with 50 mL/min dry nitrogen. Temperature axis and cell constant were calibrated using indium. Sample was initially heated to 40–50 °C below *T*_g_ to remove the moisture present with the amorphous form. Sample was then cooled back to 25 °C, followed by reheating from 25 to 200 °C temperature range (modulation amplitude ±0.3°, duration 60 seconds), with different underlying heating rates of 1, 2, 3, 4 or 5 °C/min. A baseline shift in reversing heat flow signal, concomitant with a rise in reversing heat capacity was assigned as the *T*_g_. Analysis of the data was performed using Universal^®^ Analysis 2000 (version 4.5A) software.

#### 2.2.6. Fourier Transform Infra Red (FT-IR) Spectroscopy

FT-IR spectra were recorded from 4000 to 650 cm^−1^ on Perkin Elmer Spectrum 400 spectrometer, using an attenuated total reflectance germanium crystal accessory. 32 accumulations averaged at a resolution of 4 cm^−1^ were collected. Sample was initially heated to 40–50 °C below *T*_g_ to remove the moisture present with the amorphous form. Sample was alternatively heated post *T*_g_ to determine the FT-IR spectra of corresponding recrystallized anhydrate salts (PRB HCl-AnH; PRB MES-AnH and PRB TSA-AnH). Each sample was analyzed at ambient temperature. Analysis was performed in triplicate and for each measurement; a fresh aliquot of sample was used. Data analysis was performed using Spectrum^®^ v3.02.01 (version 3.02.01) software.

## 3. Results and Discussion

### 3.1. Characterization of Amorphous Forms

**Figure 2.**
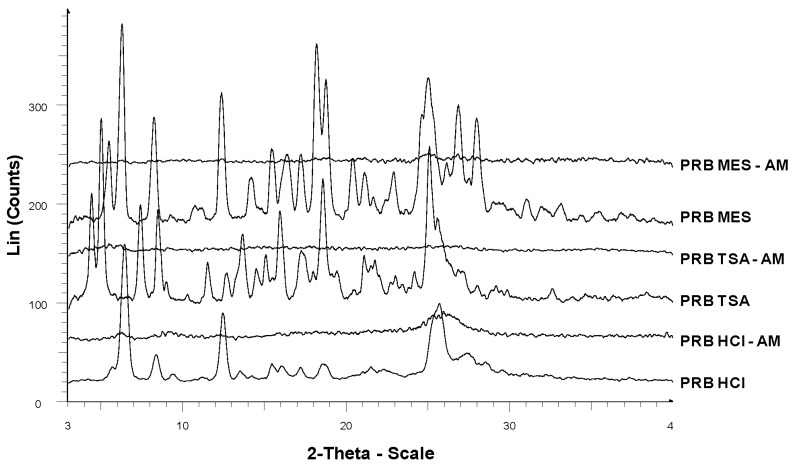
PXRD overlay of amorphous and crystalline prazosin salts. Amorphous salts show halo pattern in the PXRD diffractograms, unlike sharp peaks of crystalline salts.

Spray drying successfully generated amorphous prazosin hydrochloride (PRB HCl-AM), prazosin mesylate (PRB MES-AM) and prazosin tosylate salt (PRB TSA-AM), as indicated by absence of birefringence in polarized light microscopy and a halo pattern in PXRD diffractogram, characteristic of their amorphous nature (Figure 2).

### 3.2. Effect of Counterion on Glass Transition of Prazosin Salts

**Figure 3.**
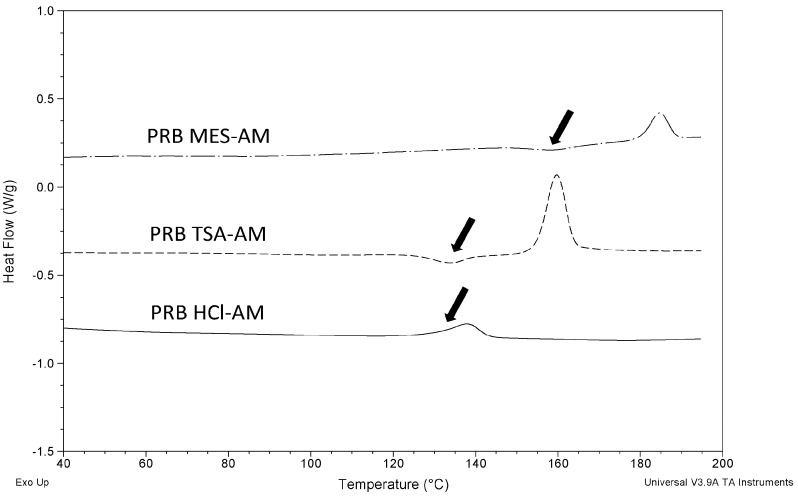
Heating curves for prazosin salts (3 K/min). Arrows denote the position of respective *T*_g_. PRB HCl-AM showed overlapping *T*_g_ and recrystallization.

**Figure 4.**
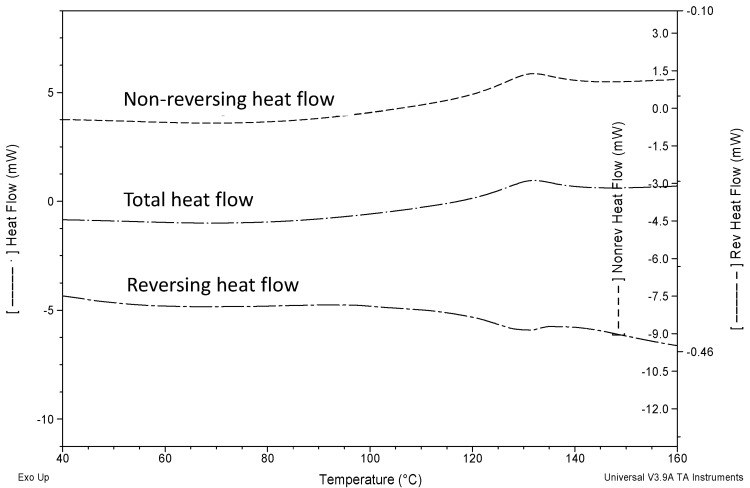
MDSC heating curve for PRB HCl-AM (3 K/min), showing reversing and non reversing heat flow. Reversing heat flow showed *T*_g_, concomitant with recrystallization event observed in the non-reversing heat flow in the same temperature range.

*T*_g_ of amorphous salts were determined using MDSC. PRB TSA-AM and PRB MES-AM showed *T*_g_ followed by the recrystallization event, and finally the melting of recrystallized anhydrate salts along with degradation (Figures 3 and 4). In contrast, PRB HCl-AM showed *T*_g_ concomitant with the recrystallization event, and followed by the melting of recrystallized anhydrate hydrochloride salt along with degradation.

Figure 4 show the total heat flow, reversing and non-reversing heat flow signals of PRB HCl-AM. Reversing heat flow signal of PRB HCl-AM show *T*_g_, occurring concomitantly with the recrystallization event, as indicated by the non-reversing heat flow signal. *T*_g_ of prazosin salts (at 3 K/min heating rate) followed the order: PRB MES-AM (433.4 K) > PRB TSA-AM (400.6 K) ∼ PRB HCl-AM (399.7 K).

Density Functional based concepts are reported for studying various molecular interactions including chemical reaction and physical attraction. DFT concepts are capable of characterizing the inherent tendency of interaction of a molecule interacts with other systems, due to the intrinsic nature of the involved functions [7,11-15]. Density functional theory (DFT) has been used to explain the intermolecular interactions affecting *T*_g_ of amorphous salts [4,5]. DFT considers three energy factors, contributing towards the *T_g_* behavior. The first is the electrostatic factor, which is dominant for ionic molecules, and is of major importance for salts. Second factor refers to covalent bonding, which emerge from the sharing of electrons. Polarization contribution is the third factor that arises due to instantaneous electron movement. These contributors are assessed in terms of DFT parameters, namely the hardness parameters, chemical potential and electrophilicity index. Hardness η represents the energy gap between ionization potential (I) and electron affinity (A), and is given by the equation 1:

(1) η=I−A

Chemical potential and electrophilicity index indicate contribution of covalent and polarization interactions [13,16]. Chemical potential μ is given by equation 2:

(2) $$\mu =-\frac{\text{I}+\text{A}}{2}$$

Electrophilicity index ω denotes the charge transfer interactions for an intermolecular process [13], and is given by the equation 3:

(3) $$\omega =\frac{\mu^{2}}{2\eta }$$

A high electrophilicity index shows a tendency to form strong covalent and polarization interactions with other similar molecules.

Table 1 shows the DFT parameters for prazosin salts. Hardness parameter η followed the trend: PRB HCl-AM > PRB MES-AM > PRB TSA-AM. In contrast, electrophilicity index ω followed the trend: PRB MES-AM ∼ PRB HCl-AM > PRB TSA-AM. It is expected that the value of these descriptors, which correspond to an increased ability to form ionic and polarization interactions, lead to a higher value of *T_g_* [4]. On the basis of DFT parameters, the observed trend in *T_g_* therefore should have been PRB HCl-AM > PRB MES-AM > PRB TSA-AM.

*T*_g_ for PRB MES-AM and PRB TSA-AM is in accordance with this hypothesis. Intermolecular force of interactions in PRB MES-AM are stronger (as indicated by higher η and ω values respectively), compared to that in PRB TSA-AM. However, the observation of PRB HCl-AM, having *T*_g_ lower than PRB MES-AM, and similar to PRB TSA-AM, is counterintuitive, thus requiring further investigation.

**Figure 5.**
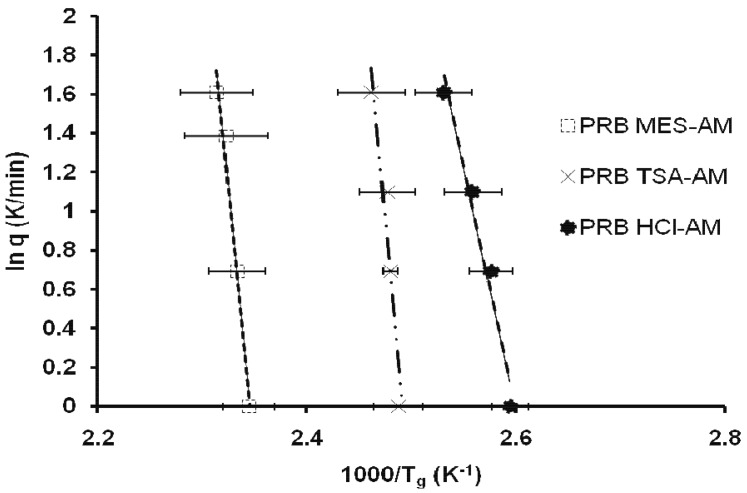
Plot of ln *q* (K/min) *vs.* inverse of *T*_g_ (K^−1^) for amorphous prazosin salts.

Apart from the ionic and covalent interactions, hydrogen bonding, which has characteristics of both ionic and covalent interactions, also affects *T*_g_ [17-19]. Ionic interactions in prazosin salts take place predominantly at the ionized N-1 ring nitrogen of prazosin, with the corresponding counterion. Moreover, the two hydrogen atoms of the free exocyclic amino group as well as N-1 hydrogen are involved in extensive intermolecular hydrogen bonding with the neighbouring counterion as well as oxygen of the furan side chain [20]. Effect of hydrogen bonding on the strength of glass against physical/chemical modification was compared in terms of apparent activation energy (*E*_a_) near *T*_g_. *T*_g_ values at different heating rates of 1, 2, 3, 4 and 5 °C/min were used to calculate E_a_ for molecular mobility, using the equation 4:

(4) $$\ln q=-E_{a}/\text{R}T$$

where q is the applied heating rate, R is universal gas constant and T corresponds to *T*_g_ measured at respective heating rates. Linear curves with regression value of 0.90–0.99 were obtained for all prazosin salts. Slope of the line gave *E*_a_ (kJ/mol) for molecular mobility of amorphous prazosin salts (Table 1; Figure 5). *E*_a_ of prazosin salts followed the trend: PRB TSA-AM (490.7) > PRB MES-AM (448.5) > PRB HCl-AM (206.1). Lower *E*_a_ for PRB HCl-AM suggested weaker kinetic barrier for conversion to the supercooled state and subsequent recrystallization.

Gunawan *et al.* studied the structural relaxation of acetaminophen glass and demonstrated that in order for molecules to diffuse; hydrogen bonds must be broken, followed by formation of new hydrogen bonds between them. It was concluded that the strength of hydrogen bonding in molecules significantly impacts the molecular mobility and hence further the tendency to recrystallize [21]. Difference in hydrogen bonding strength between the amorphous and recrystallized form further indicate the kinetic barrier to glass transition and subsequent recrystallization. Relatively stronger hydrogen bonding in amorphous form compared to the recrystallized form would therefore require higher E_a_ for molecular mobility, and *vice versa*.

It was observed for prazosin salts that the hydrogen bonding is stronger in PRB HCl AnH, compared to PRB HCl-AM (discussed in detail in section 3.2). In contrast, hydrogen bonding in sulfonate salts is similar in both amorphous and the crystalline state. Therefore, lower E_a_ was required for molecular mobility of PRB HCl-AM, compared to that for PRB TSA-AM and PRB MES-AM.

### 3.3. Effect of Counterion on Recrystallization Behavior of Prazosin Salts

Methods used to generate the amorphous form been known to affect the recrystallization tendency of the amorphous form. Surana *et al.* investigated the properties of amorphous trehalose prepared by freeze-drying, spray drying, dehydration, and melt quenching. It was observed that the processing conditions did not impact the glass transition temperature and fragility, but the properties like enthalpy relaxation, recrystallization tendencies, and water sorption were greatly altered [22]. In our study involving prazosin salts, amorphous forms were generated by an optimized spray drying protocol, thus obviating the effect of sample preparation for the comparative assessment of glass transition and recrystallization behavior.

Recrystallization tendency of prazosin salts was determined using the reduced crystallization temperature (RCT). RCT represents a normalized measure of how far above *T*_g_ a compound must be heated, before spontaneous crystallization can occur. It is a useful method to compare the recrystallization tendency of compounds with different *T*_g_, and is represented by equation 5 [23].

(5) $$\text{RCT}=\left(T_{\text{c}}-T_{\text{g}}\right)/\left(T_{\text{m}}-T_{\text{g}}\right)$$

where *T*_c_ is the recrystallization temperature and *T*_m_ is the melting temperature. Crystallization tendency of PRB MES-AM and PRB TSA-AM is similar, owing to similar values of RCT. In contrast, crystallization tendency of PRB HCl-AM is significantly higher, as indicated by a very low value of RCT (Table 2).

Recrystallization from amorphous state is a complex phenomenon, affected by a number of variables. Since crystallization involves nucleation as well as crystal growth, factors affecting one or both processes, may impact the overall recrystallization behavior. These include the thermodynamic (e.g., free energy difference between the crystalline and amorphous state), kinetic (molecular mobility), or molecular level interactions (e.g., hydrogen bonding and ion-dipole interactions) [24]. Influence of thermodynamic parameters for prazosin salts could not be determined due to their degradation along with melting. Contribution of kinetic parameters was determined by the heating rate dependence of *T*_g_, which indicates kinetic barrier to glass transition and subsequent recrystallization (discussed in section 3.1). E_a_ of prazosin salts followed the trend: PRB TSA-AM (490.7) > PRB MES-AM (448.5) > PRB HCl-AM (206.1). Lower E_a_ for PRB HCl-AM suggested weaker kinetic barrier for conversion to supercooled state and subsequent recrystallization.

Effect of molecular level interactions was further evaluated by comparing the hydrogen bonding interactions in amorphous and crystalline state. Intermolecular hydrogen bonding in prazosin salts predominantly involve interaction of the hydrogen atoms of the exocyclic amino group or N-1 quinazoline nitrogen with the neighbouring counterion as well as oxygen of the furan ring (Figure 1) [20]. Intermolecular hydrogen bonding exists in both amorphous and the crystalline state. However, their relative strength indicates the tendency to undergo *T*_g_ and subsequent recrystallization. Stronger hydrogen bonding strength in amorphous form, compared to the crystalline form tends to favor the crystallization process, and *vice versa*.

**Figure 6.**
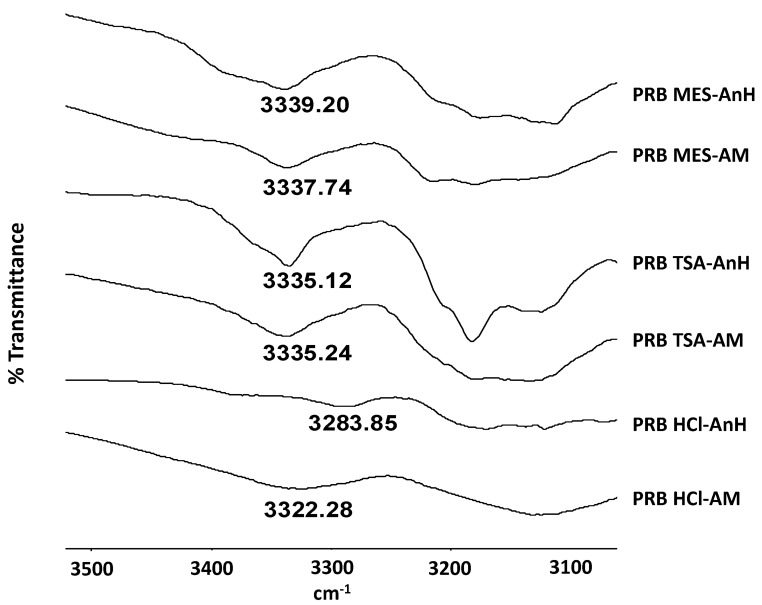
FT-IR peaks of PRB HCl-AnH, PRB HCl-AM, PRB TSA-AnH, PRB TSA-AM, PRB MES-AnH and PRB MES-AM salts.

Hydrogen bonding strength of prazosin salts was assessed in terms of NH stretching peak position (Figure 6; Table 3). Recrystallization of PRB HCl-AM (NH peak: 3322.28 cm^−1^) to PRB HCl AnH (NH peak: 3283.85 cm^−1^) led to shift in NH peak to higher wavenumber, indicating strengthening of hydrogen bonding interactions during recrystallization. Therefore, it can be speculated that recrystallization of PRB HCl-AM is thermodynamically favored. In contrast, for sulfonate salts, no significant shift in NH stretching peak was observed, indicative of similar hydrogen bonding strength in both amorphous and the crystalline state. This can be correlated to a higher E_a_ observed for PRB TSA-AM and PRB MES-AM, compared to PRB HCl-AM, for molecular mobility at *T*_g_.

Tang *et al.* studied the hydrogen bonding patterns of amorphous and crystalline nifedipine and felodipine by Raman and IR spectroscopy [25]. Nifedipine showed stronger hydrogen bonding in crystalline state than in the amorphous form. In contrast, felodipine exhibited stronger hydrogen bonding in amorphous form. Difference in hydrogen bonding strength between the crystalline and amorphous forms was observed to affect the crystallization tendency. During crystallization of nifedipine, hydrogen bonding strength increased, thus favoring the crystallization process. In contrast, felodipine crystallized at the expense of reduction in hydrogen bonding strength. Therefore, higher E_a_ was observed for felodipine relative to nifedipine, to weaken hydrogen bonding prior to crystallization.

In our study, PRB HCl-AM showed faster recrystallization due to favored hydrogen bond strengthening during recrystallization to PRB HCl. In contrast, no significant change in hydrogen bond strength was observed in PRB TSA-AM and PRB MES-AM, corresponding to their recrystallized anhydrate forms. This led to slower recrystallization of PRB TSA-AM and PRB MES-AM from the amorphous form.

### 3.4. Practical Relevance of the Study

The importance of this work lies in its utility for selection of a stable amorphous salt form, suitable for pharmaceutical development. *T*_g_ and recrystallization behavior are the prime indicators of physical and/or chemical stability of an amorphous form. It is preferred to have an amorphous salt form with high *T*_g_ as well as low recrystallization tendency.

Generally, hydrochloride salt of a drug is preferred to prepare amorphous salts, owing to its higher *T*_g_ [4-7]. However, in this study, amorphous prazosin hydrochloride salt had a lower *T*_g_ and higher recrystallization tendency, compared to amorphous prazosin sulfonate salts. This study therefore stresses upon a careful screening for *T*_g_ and recrystallization tendency of amorphous salts, than mere empirical selection of a particular amorphous salt form. Although this study has been performed using acidic counterions, it would be interesting to confirm these findings on amorphous salts of basic drugs.

## 4. Conclusions

This study evaluated the effect of counterion on the glass transition and recrystallization temperature of prazosin salts. Amorphous prazosin hydrochloride salt showed lower glass transition temperature and a higher recrystallization tendency, compared to amorphous prazosin mesylate and prazosin tosylate. This counterintuitive observation was explained in terms of lower activation energy for molecular mobility of amorphous prazosin hydrochloride, compared to amorphous prazosin mesylate and prazosin tosylate salts. The observation was further correlated to a lower hydrogen bonding strength of amorphous prazosin hydrochloride salt, compared to its recrystallized counterpart. In contrast, hydrogen bonding strength in sulfonate salts was similar in both amorphous and the recrystallized state. This study stresses the need to screen for glass transition and recrystallization temperature, to select an optimal amorphous drug salt during early drug development phase.

## Figures and Tables

**Table 1.** Selected molecular descriptors for the studied counterions calculated using DFT [7].

**Counterion**	**A (eV)**	**I (eV)**	**η (eV)**	**μ (eV)**	**ω (eV)**
HCl	−0.49	12.76	13.25	−6.14	1.42
TSA	0.30	9.12	8.82	−4.71	1.26
MES	0.32	10.77	10.45	−5.54	1.47

HCl: Hydrochloric acid; TSA: Toluenesulfonic acid; MES: Methanesulfonic acid; A: electron affinity. I: ionization potential; η: hardness. μ: electronic chemical potential. ω: electrophilicity index

**Table 2.** Thermal characterization of prazosin salts.

**Salt**	***T*_m_ (K)^#^**	***T*_g_ (K)^#^**	***T*_m_/*T*_g_**	***E*_a_ (kJ/mol)**	*T*_c_ (K)*	**RCT^$^**
PRB MES-AM	540.13	433.4	1.25	448.5	453.0	0.16
PRB TSA-AM	573.13	400.6	1.45	490.7	428.0	0.18
PRB HCl-AM	545.13	399.7	1.38	206.1	402.0	0.01

# heating rate: 3 K/min; *T*_m_: melting temperature (onset); *T*_g_: glass transition (midpoint-inflection);

$ reduced crystallization temperature.

**Table 3.** FT-IR peaks for prazosin salts.

**Sample**	**FT-IR peak (cm^−1^)**	**Peak shift (cm^−1^)^@^**	**Effect on hydrogen bonding**
PRB HCl-AM PRB HCl-AnH	3322.28 3283.85	−38.43	Stronger hydrogen bonding in crystalline state
PRB TSA-AM PRB TSA-AnH	3335.24 3335.12	−0.01	no significant change
PRB MES-AM PRB MES-AnH	3337.74 3339.20	1.46	no significant change

@ peak shift = wavenumber (amorphous) – wavenumber (crystalline)
